# Anatomy, Biomechanics, and Loads of the Wrist Joint

**DOI:** 10.3390/life12020188

**Published:** 2022-01-27

**Authors:** Jörg Eschweiler, Jianzhang Li, Valentin Quack, Björn Rath, Alice Baroncini, Frank Hildebrand, Filippo Migliorini

**Affiliations:** 1 Department of Orthopaedics, Trauma and Reconstructive Surgery, RWTH Aachen University Hospital, Pauwelsstraße 30, 52074 Aachen, Germany; jli@ukaachen.de (J.L.); vquack@ukaachen.de (V.Q.); fhildebrand@ukaachen.de (F.H.); fmigliorini@ukaachen.de (F.M.); 2 Department of Orthopaedic Surgery, Klinikum Wels-Grieskirchen, 4600 Wels, Austria; Bjoern.Rath@klinikum-wegr.at; 3 Department of Orthopaedic and Trauma Surgery, Eifelklinik St. Brigida, Kammerbruchstraße 8, 52152 Simmerath, Germany; alice.baroncini@artemed.de

**Keywords:** wrist, biomechanics, loads, anatomy

## Abstract

The wrist is by far the most differentiated section of the musculoskeletal system. The spectrum of wrist injuries ranges from minor injuries to complex traumas with simultaneous loss of functions, resulting in enormous economic costs. A proper understanding of the anatomy and biomechanics is essential for effective treatment, whether conservative or surgical; this applies to the wrist no less than to other parts of the human body. Here; information on the wrist anatomy; kinematics; and biomechanical behavior is presented, commencing with a brief explanation of the structure of its hard and soft tissues. Eight carpal bones in combination with two forearm bones (radius and ulna) construct the wrist joint. The motion of the wrist joint is initiated by the muscles of the forearm, and strong and short ligaments ensure the stability of the wrist. All of these components are essential to bringing functions to the wrist joint because these structures allow wrist mobility and sustainability. In addition, the kinematics of the wrist joint is presented and different biomechanical model approaches. The therapeutic (surgical) restoration of the balance between the load–bearing capacity and the actual stress on a joint is the prerequisite for a lifelong and trouble-free function of a joint. Regarding the complex clinical problems, however, a valid biomechanical wrist joint model would be necessary as assistance, to improve the success of systematized therapies based on computer–aided model–based planning and intervention.

## 1. Introduction

The wrist is involved in many functional activities. It is exposed to a high number of traumatic injuries and degenerative diseases [1]. The wrist is a complex joint that allows movement of the hand in multiple directions relative to the forearm [2].

Knowledge of biomechanical behavior is important for a basic science perspective and also from a clinical point of view. To understand and treat wrist pathologies, it is essential to understand the carpal biomechanics, including the function of the soft tissue, e.g., ligaments [3,4,5]. It is important to understand the basic science, and furthermore, the clinical relevance of functional kinematics of the wrist joint—defined as those motions that are necessary to carry out high-demand activities of daily living [6]. Also, the understanding of physiological and pathological biomechanical behavior is important to investigate the cause and effects of injuries and surgical repair [5]. Current management of wrist ailments often lead to unsatisfying outcomes [1]. One of the reasons for this is the difficulty of thoroughly analyzing the behavior of the disease due to its complexity [1].

Biomechanical models can be implemented as clinical aids for the evaluation of the wrist joint of the patient before, and post-surgery. Up to date, the surgical planning in clinical practice is based solely on the status of the individual wrist and primarily to its radiographic appearance. Furthermore, secondarily to additional image techniques up to an arthroscopic intervention for a final diagnosis. For validation the underlying models, information derived from biomechanical in vivo studies are needed and used. This information are also important for the development of surgical simulation, and the planning tools for wrist joint surgery or for the implantation of an artificial joint/a wrist joint endoprosthesis. The simulation and planning tools are used for optimizing, e.g., to decrease the resulting joint loading, to prevent the progression of pathologic changes to the wrist (e.g., osteoarthritic changes), and to increase the longevity of an endoprosthesis. Personalized modeling, biomechanical simulation, and load analysis of implants, and their boundary conditions have been advocated. The simulation of such a complex joint system is challenging, complex, and thus has received little attention [1,7,8].

This article reviews certain basic features of wrist anatomy and the complex structure of the wrist joint. This includes information that is important to understand physiological wrist kinematics, respectively, and applies them to a review of wrist joint biomechanics and load transfer.

## 2. Anatomy of the Wrist Joint

### 2.1. Bones

**Figure 1:**
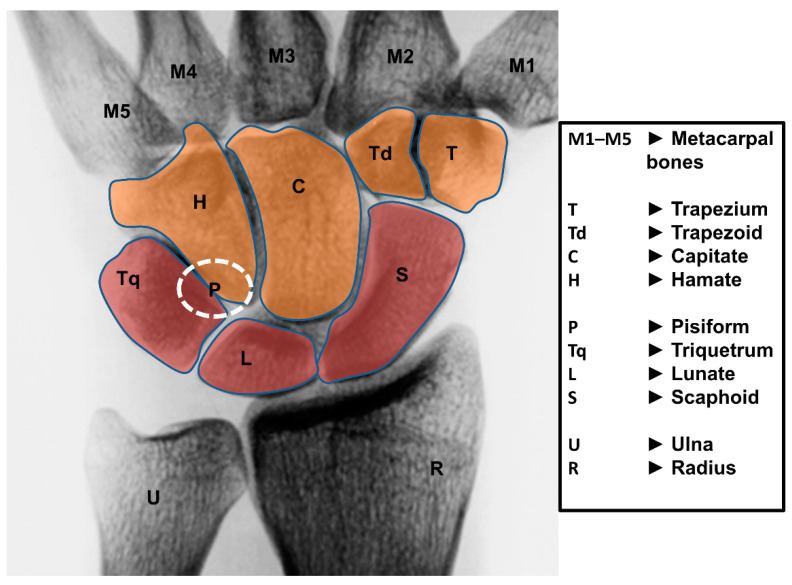
Bones of the wrist from dorsal. The orange-colored bones are the DCR, the red colored bones belong to the PCR. The pisiform (P) is just indicated because it is positioned on the palmar side.

The wrist joint is a diarthrodial joint and is built up of eight unique carpal bones. They are interposed between the forearm (radius and ulna) and the five metacarpal bones (Figure 1). The wrist is composed of two rows of carpal bones: the proximal carpal row (PCR) includes from radial to ulnar the scaphoid, lunate, triquetrum, and pisiform; the distal carpal row (DCR) includes from radial to ulnar the trapezium, trapezoid, capitate, and hamate.

The bones of the DCR are tightly bound to each other via strong ligaments, and they creating essentially a single functional unit [4,9]. The nearly rigid ligamentous connection of the DCR to the basis of the metacarpal bones allows considering the DCR functionally as part of a unit that moves in response to the muscle forces of the forearm [9]. The bones of the wrist are rigidly attached to each other by a series of ligaments resulting in limited movement between the bones [9].

The PCR behaves differently in comparison to the DCR. There exists a significant motion between adjacent bones, and additionally, the entire row moves in generally the same direction. The PCR moves in combination with the DCR during wrist flexion and extension (FE) but continues to experience FE during wrist radial and ulnar deviation (RUD). The PCR is described and acts as an intercalated segment between the radius and the DCR [9,10,11,12].

None of the muscles acting on the wrist are attached to the PCR [13]. All of the tendons that influence wrist motion insert distally. The PCR motion depends entirely on mechanical forces from their surrounding articulations [4,14]. Thus, wrist motion in any plane must be initiated at the DCR. Motion in the PCR begins only when the extrinsic ligaments crossing the midcarpal joint become taut and the force applied to the PCR becomes greater than the frictional forces of the intervening articular segments and the resistance of the antagonistic muscular forces. The wrist relies on the unique bone morphology, their unique interaction with neighboring bones, and their extrinsic and intrinsic ligaments [15].

The triangular fibrocartilage complex (TFCC) is a load–bearing structure. The TFCC is located on the medial part of the wrist between the lunate, triquetrum, and ulnar head. It is built up of a triangular fibrocartilage articular disc, in addition to the ulnomeniscal homologue, ulnar collateral ligament, dorsal and palmar radio-ulnar ligaments, the base of the extensor carpi ulnaris sheath, and the ulnolunate and ulnotriquetral parts of the palmar ulnocarpal ligament. Its function is to act as a stabilizer for the ulnar aspect. Furthermore, the TFCC prevents ulnocarpal abutment by transmitting and distributing axial load from the carpus to the ulna. It facilitates the movements at the wrist.

### 2.2. Ligaments

#### 2.2.1. General

The wrist ligamentous structure is extremely complex, comprising in sum 33 intra-articular and intra–capsular ligaments [16]. In the literature, the wrist joint ligaments are quite variably described. This can lead to a confusion regarding their anatomy. The different descriptions, classifications, and nomenclatures increase the complexity of understanding this region [17]. Different articles about the anatomy and function of the carpal ligaments have been published (e.g., [2,12,18,19,20,21,22,23]), recent detailed information has further elucidated the ligamentous wrist anatomy [4].

The ligaments play a crucial role in guiding and constraining carpal bone motion during the overall movements of the hand. They include extrinsic and intrinsic ligaments where the extrinsic ligaments connect the carpal bones to the radius or metacarpals and include volar and dorsal ligaments. The intrinsic ligaments originate and are inserted on the different carpal bones.

#### 2.2.2. Extrinsic Carpal Ligaments

The carpus is supported by a ligamentous system that prevents unidirectional migration of the carpal segment [24]. The extrinsic ligamentous apparatus (Table 1) courses between the carpal bones and the radius or the metacarpals. In carpal kinematics, the functional role of the extrinsic wrist ligaments is still poorly understood [15,25]. The extrinsic wrist ligaments include the dorsal intercarpal (DIC) ligament, dorsal radiocarpal (DRC) ligament, radioscaphocapitate (RSC) ligament, long radiolunate (LRL) ligament, short radiolunate (SRL) ligament, ulnolunate, and ulnocapitate ligament [15]. The extrinsic wrist ligaments are part of a confluence of wrist ligaments. This includes that different regions of the ligaments differentially strained depending on the direction of the motion [6,26].

#### 2.2.3. Intrinsic Carpal Ligaments

The intrinsic ligaments (Table 2) originate and insert within the carpus [24]. Most of the carpal bones are directly attached to their neighboring bones through interosseous ligaments. There exists no ligamentous connections between the lunate and capitate. Due to the mobility of the PCR, the focus will be on the ligaments connecting the bones of the PCR. The PCR bones are attached by the scapholunate interosseous ligament (SLIL) and the lunotriquetral interosseous ligament (LTIL). The two ligaments are C–shaped, leaving the distal aspect of these bones available for articulation with the DCR [27]. The most important intrinsic ligaments are the SLIL and the LTIL.

### 2.3. Muscles

The muscles acting on the wrist joint are situated within the forearm. Only the muscles tendon crossing the wrist joint and inserting on the hand/ fingers. There are no tendons directly attached to the carpus and PCR [22,24]. The muscles on the dorsal side of the forearm act to extend, and the muscles on the palmar side act to flex the wrist. Wrist bone motion depends entirely on mechanical forces from their surrounding articulations [4,22]. Physiological carpal biomechanics rely on the interactions between the ligaments and the morphology of the carpal bones [5]. Wrist bone motion is complex and occurs in three dimensions. Many wrist problems are the result of an alteration of intercarpal motion [30]. Currently, intercarpal motion remains incompletely defined [5,30,31].

**Figure 2:**
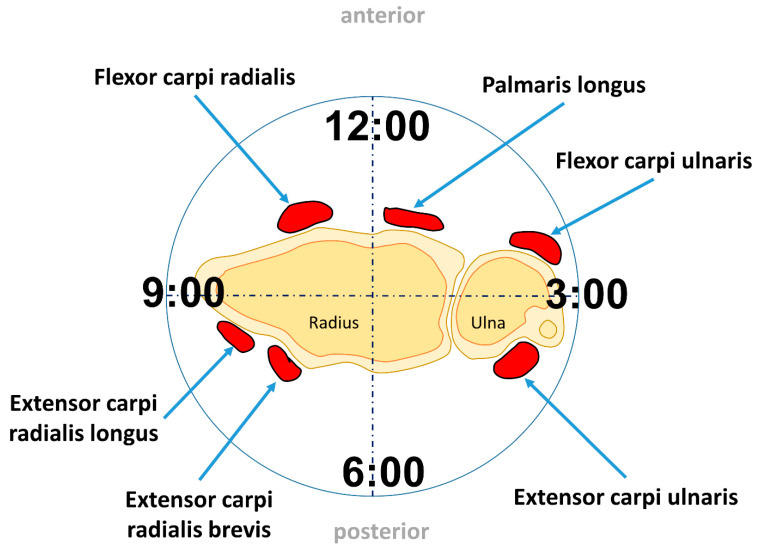
Wrist muscles position from a distal to proximal view.

Of the numerous muscles in the forearm, six muscles are inserted at the carpal bones (at the DCR) or the base of the metacarpal bones (Figure 2).

These six muscles contribute to moments about the FE and RU axes [32]. They are dedicated solely to the wrist and include on the anterior side the flexor carpi radialis (FCR), flexor carpi ulnaris (FCU), and the palmaris longus (PL).

On the posterior side are three primary wrist extensor muscles: the extensor carpi radialis longus (ECRL), extensor carpi radialis brevis (ECRB), and extensor carpi ulnaris (ECU) (Table 3). They have larger moment arms about the wrist axes [33]. The flexion is mainly produced by the PL, FCU, and FCR. The motion is supported by the flexor digitorum superficialis muscle. The extension is mainly produced by the ECRL, ECRB, and ECU. They will be assisted from the extensor digitorum muscle. The adduction is produced by the ECU and FCU. The abduction is produced by the FCR, ECRL, and ECRB. It is supported by the abductor pollicis longus muscle.

## 3. Wrist Motion

### 3.1. Overall Motion

In general, the wrist is approximated as a two degree of freedom (DOF) universal joint. Traditionally, the motion has been defined in terms of two orthogonal anatomical axes: the FE–axis and RUD–axis [6]. The motion of the hand to the forearm can be described via two DOF rather than the usual six DOF that have been described for the traditional kinematic analysis [32,34]. It is possible, in case of a fully pronated right wrist, mapping the directions of wrist rotation onto a clock face where FE occur at 6:00 and 12:00, RUD occur at 9:00 and 3:00 (Figure 2) [6].

**Figure 3:**
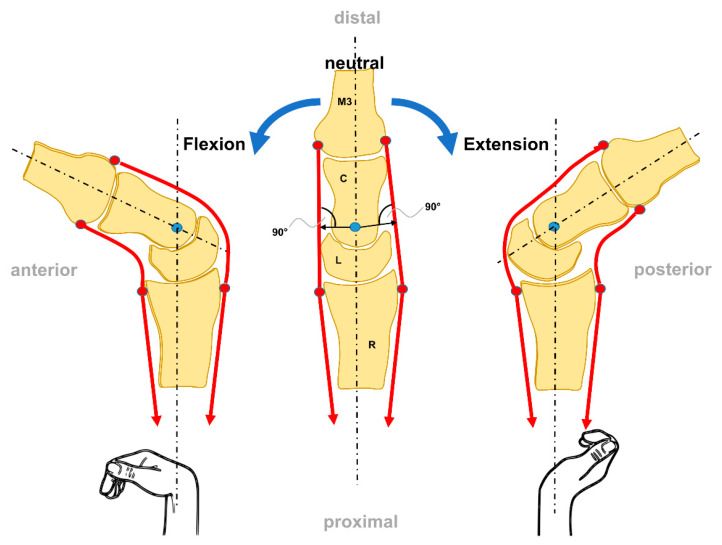
The FE movement from a sagittal view (modified after [35]).

The DCR bones behave as a unit that is moved by off-center forces. This is similar to the beam of a scale moving around an instantaneous axis through the head of the capitate [14]. FE movements (sagittal plane) are evenly distributed between the radiocarpal and midcarpal joints (Figure 3).

**Figure 4:**
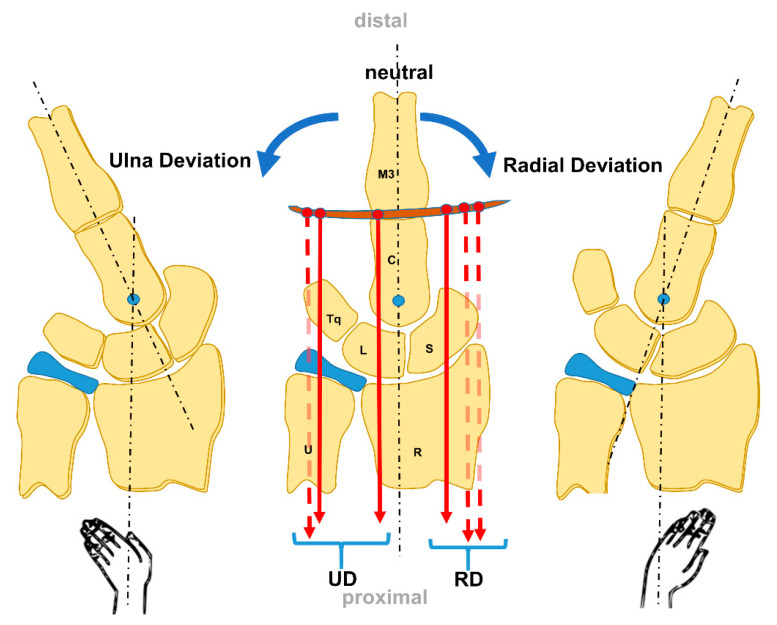
The RUD Movement from a palmar view (modified after [35]).

The RUD movements (frontal plane) occur through the midcarpal joint [14] (Figure 4). Functional motion arcs for activities of daily living are 5–10° to 30–35° FE, and 10° to 15° of RUD [14].

To complete the motion behavior, wrist motion during daily activities replicates the dart thrower’s motion, from radial deviation and extension to ulnar deviation and flexion. Dart-thrower’s arc is one of the widely used wrist motion during daily activities [14]. 

There exists still a controversy about the existence of a “center of rotation” of the wrist. If one exists it will be found in the head of the capitate, for both FE and RUD [3,36,37,38,39].

### 3.2. Specific Wrist Bone Motion

Wrist motion is a complicated interaction of seven carpal bones excluding the pisiform which is a sesamoid bone and the forearm. Each of them with a separate motion axis yet is interdependent on the position of adjacent carpal components and the carpal alignment with the distal radius [40,41]. In general, there is appreciable motion between the radius and the PCR, less motion between the PCR and DCR, and no motion between the DCR and the metacarpals [20].

From a functional standpoint, carpal motion varies within each row, particularly within the PCR [2]. The DCR bones act as a functional unit. Under an axial load, the DCR tends to rotate into pronation, the scaphoid into flexion, and the triquetrum toward extension [42]. This unit also includes the metacarpals because of the interlocking of the articular surfaces and the dense ligamentous connections between the bones of the DCR and the bases of the metacarpals. In case of the third metacarpal flexes or extends, the DRC moves similarly [2]. 

The PCR bones come along with a unique motion pattern. The PCR moves together with a higher motion between the individual bones than between the DCR [41]. The lunate shows the least range of motion (ROM), followed by the triquetrum and scaphoid. The PCR bones flex during global wrist flexion and extend during global wrist extension. In case of global wrist extension, the scaphoid shows the tendency to supinate and the lunate to pronate. A reverse phenomenon occurs during global wrist flexion. In case of global wrist RUD, the PCR bones demonstrate a unique motion behaviour that is best described as reciprocal [2,43]. Radial deviation leads to palmar flexion and secondarily and variably counter−rotation of the PCR bones toward the ulnar margin of the wrist. Ulnar deviation leads to a principal extension and secondarily and variably counter rotation toward the radial margin of the wrist. The same longitudinal motion behavior take place between the PCR bones during RUD as occurs during wrist FE. Therefore, the PCR bones accommodate wrist RUD by palmarflexing and dorsiflexing, respectively [2,44].

Patterson et al. reported on carpal kinematics during simulated active and passive cadaveric wrist motion using an optical tracking system [45]. They described that there were no significant differences in carpal bone motion (FE and RUD) when the wrist was moved actively via the extensor and flexor tendons or passively, with a constant force applied to the tendons [45]. They concluded that carpal bone kinematics in a healthy joint is similar in active and passive wrist motion [45]. A comprehensive understanding of normal carpal architecture and functions is essential for prevention of pathological changes in the wrist [6,46].

## 4. Biomechanics of the Wrist Joint

### 4.1. Models and Theories of the Wrist Joint Biomechanics

To understand and treat wrist injuries and degenerative changes, it is essential to understand the carpal biomechanics [3,4,5]. Techniques for quantifying carpal bone kinematics as a function of wrist position have dramatically evolved, and this evolution has been linked to the development of biomechanical models and theories [6]. A good clinical understanding especially of the kinematics of the carpal bones during wrist motion is necessary to effectively diagnose and treat wrist injuries [3,47,48]. Multiple explanations for intercarpal motion have been suggested, however the mechanisms for the degree and direction of motion of each carpal bone, that make up the two carpal rows, during motion in each of the planes, remain controversial [9,49]. Different prevailing theories have been used to characterize carpal kinematics.

#### 4.1.1. Row-Theory

**Figure 5:**
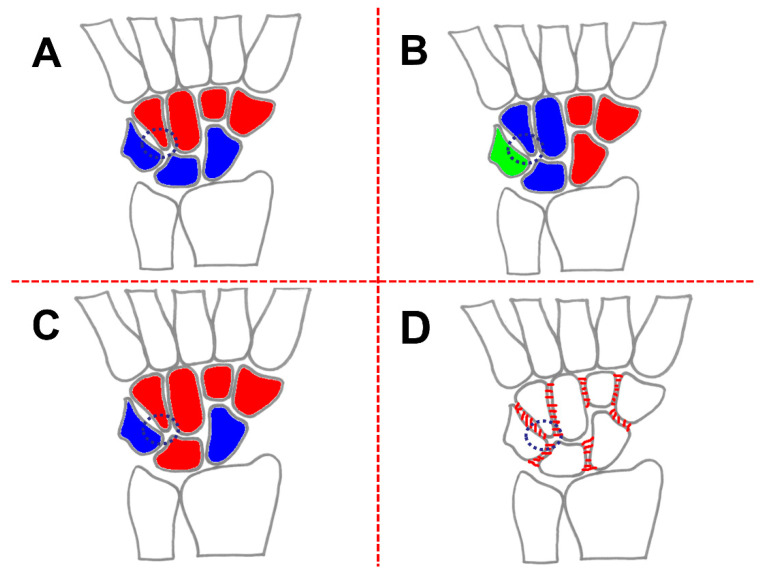
Different theories/models of the wrist joint (**A**) Row theory; (**B**) Column-theory; (**C**) Row-column-theory; (**D**) Ring-model (modified after [50]).

Based on their kinematic behavior during global wrist motion [4,9] and also on anatomic investigations [24], the carpal bones are traditionally clustered into a PCR and DCR (Figure 5A). 

The PCR can be described as an intercalated segment [9]. No tendons are inserted upon them [9]. 

The DCR are rigidly bound to one another via stout ligaments, and the motion between them can be considered negligible [9]. The DCR bones moves with the hand, to which it is attached firmly [22]. Their motion behavior depends on the response of the musculotendinous forces of the forearm [9].

#### 4.1.2. Column-Theory

The “column theory”, introduced by Navarro in 1921 [51], describes the carpus as a series of three longitudinal ulnar columns [10,49].

Navarro postulated that there exists a central, or FE column. The FE column includes the lunate, the capitate, and the hamate; the lateral column, or mobile column, includes the scaphoid, the trapezium, and the trapezoid; and medial column, or rotation column, consists of the triquetrum and pisiform [10,22,49]. The columnar carpus is demonstrated in Figure 5B. Furthermore, in this theory, the scaphoid and the triquetrum are considered independent parts of a complex carpal mechanism [22]. The triquetrum is important, particularly its articulation with the ulnar bony facet of the hamate, which is shaped to favor helicoidal or rotatory movements [22,49].

#### 4.1.3. Row-Column-Theory

The result of Taleisnik´s anatomy sections (17 human preparations) was a modification of the model theory of Navarro (Navarro, 1921) (Figure 5C) [22]. Taleisnik added the trapezium and trapezoid to the central column and eliminated the pisiform from the medial column [10].

“Perhaps we should modify Navarro’s interpretation, because the pisiform does not actually participate in carpal motion and the trapezium and trapezoid are an integral part of the distal carpal row” [22].

The mobile column is limited to the scaphoid and the rotation column to the triquetrum. Similar to in Navarro´s theory, the scaphoid and the triquetrum take an exceptional position: the scaphoid which is a lateral "mobile" column is considered to be the stabilizing link for the midcarpal joint, and the triquetrum is which is the medial or “rotation” column is thought to be the pivot point for carpal rotation [10,22,24].

In the row-column theory FE occur through the central column [10]. The RUD occur by rotation of the scaphoid laterally and the triquetrum medially [10]. 

#### 4.1.4. Ring-Model

Based on clinical observations (10 patients) as well as anatomical investigations (23 human preparations) Lichtman et al. described the so–called ring model of the carpus, with two mobile links [52] (Figure 5D). The mobile links are represented by the mobile trapezioscaphoid articulation and the rotatory triquetrohamate joint [52]. Furthermore, they included the intrinsic ligamentous apparatus in their model. Intercarpal ligaments cause the PCR to rotate as a unit [52].

The central point of this concept is the observation that RUD and FE occur reciprocally between the radiocarpal and midcarpal joints [10,52]. In detail, the ring model describes the motion behavior such as the following: movement by one row is in the opposite direction from that by the other [52]. A complete interruption at any point of the oval ring especially of the proximal carpal row results in carpal instability [10].

#### 4.1.5. Link Joint-Theory

**Figure 6:**
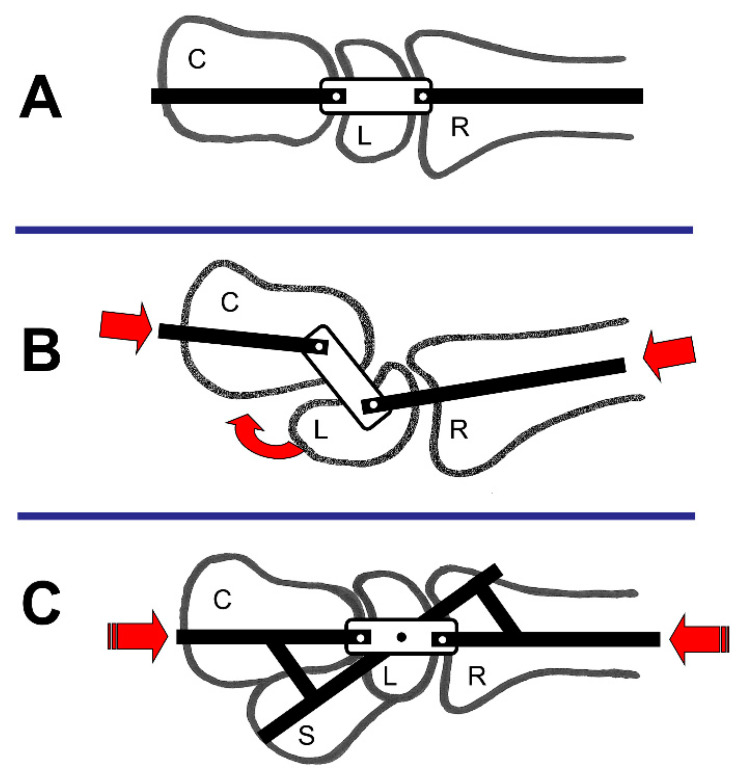
Link joint theory; S = scaphoid, L = lunate, C = Capitate, and R = Radius; (**A**) General constitution; (**B**) Load applied on the wrist; (**C**) Stabilisation of the wrist joint (modified after [10,50]).

Gilford et al. described the wrist as a link joint [53]. The wrist is similar to a link mechanism in which the radius, the PCR, and the DCR comprise the individual links [52] (Figure 6A). They mentioned that if compression force is applied, the link mechanism will collapse in a “zig–zag pattern” (Figure 6B) unless a control rod is present (Figure 6C) [52].

The scaphoid (S) links the radius (R) to the DCR (C) and provides stability against compression forces (red arrow) during wrist FE [10]. The scaphoid has been considered the control rod for the link mechanism of the wrist [52].

#### 4.1.6. Ovoid-/C-Shape-Theory

Moritomo et al. developed their “Ovoid/C-shape-theory” based on magnet resonance imaging (MRI) investigations [30]. They investigated the 3D kinematics of the midcarpal joint in the right wrists of 24 healthy volunteers via a markerless bone-registration technique. They separately investigated the kinematics during the dart-throwing motion in 12 volunteers and the kinematics during the FE motion in the other 12. For five of the 12 wrists in the dart-throwing group, MRI images were acquired in six positions (60° of radial deviation/extension to 40° of ulnar deviation/ flexion in 20° increments). For five of the 12 wrists in the FE group, MRI images were acquired in seven positions from 60° to 60° for FE. For the other seven patients in each group, three MRI scans were acquired in a neutral position and two extreme positions. 

**Figure 7:**
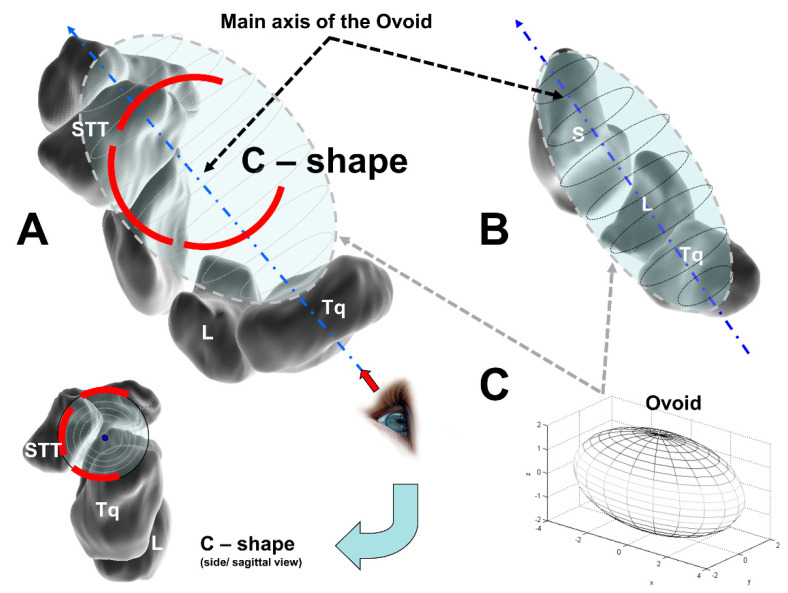
The Ovoid/C–shape theory; (**A**) L = lunate, and Tq = triquetrum, schematic of the dorsodistal view of the midcarpal ovoid with which the scaphotrapeziotrapezoid (STT) joint is in contact; (**B**) S = scaphoid, separated view of the Ovoid; (**C**) Ovoid in 3D (modified after [50]).

Their analysis showed that most of the joint surfaces of the lunocapitate and triquetrohamate joints are also part of the midcarpal ovoid. The major axis of this structure runs in a radiopalmar to an ulnodorsal direction (Figure 7A). Most of the midcarpal joint surfaces are contained within a midcarpal ovoid; the carpal bones might be moving within this volume, but they still have distinct motions relative to each other within it [30].

They postulated that midcarpal motion is the combination of the motion of three types of joint systems: (1) the uniaxial joint between the scaphoid and the DCR, (2) the biaxial and ellipsoidal joint between the lunate and triquetrum and the DCR; and (3) the intercarpal joints of the PCR [30].

Moritomo et al. advocate the use of an “Ovoid/C-shape concept” to explain the carpal self-stabilizing mechanism [30]. The 3D configuration of a line connecting the centers of the joint surfaces of the midcarpal joint can be schematized as a letter “C” entwining a midcarpal ovoid (Figure 7). On an axial radiograph of the ovoid, the midcarpal joint displays a C–shaped outline.

#### 4.1.7. Screw Vice or Clamp-Theory

**Figure 8:**
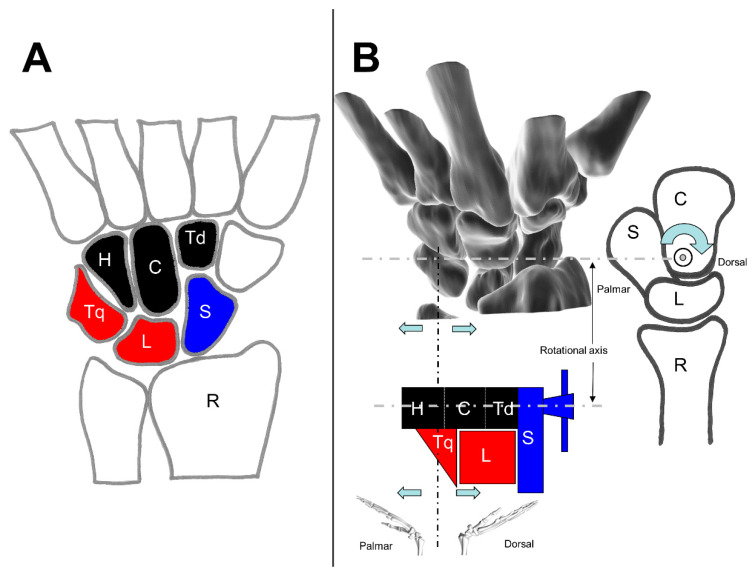
The screw clamp theory; (**A**) Overview of the wrist, S = Scaphoid, L = lunate, Tq = triquetrum, H = Hamate, C = Capitate, and Td = Trapezoid; (**B**) Schematic of the screw clamp, R = Radius (modified after [50]).

MacConaill published in 1941, based on his investigations at one human preparation, his theory about the working of the carpus as a unitary structure [54]. He divided the carpus under a functional aspect: “the carpus so defined is divisible into three masses: the navicular (scaphoid) bone; the lunate and triquetral bones together; and a distal mass formed of the hamate, capitate, and trapezoid (lesser multangular) conjointly”. [54] (Figure 8A)

The idea of this division is the separation of the scaphoid (navicular) from the other bones of the PCR: it is based upon the observed fact that this bone moves at times with the proximal and at times with the distal row [54].

MacConaill explained the procedure of the carpus is welded together in dorsiflexion. The closer packing of the proximal row is brought about by a two-stage process. “The first stage of dorsiflexion is, then, one in which the clamp is set up, or constituted, by fixing the navicular, the fixed jaw of the vice, to the distal row, which acts mechanically as the base of the vice. In the second stage, the hamate acts as a screw to pin the lunate against the fixed jaw, and to hold it there for so long as dorsiflexion is maintained” [54] (Figure 8B).

The different represented models and theories describe the motion behavior of the wrist, whereby in each case only partial aspects of the interaction behavior are considered. Arbitrary combinations of hard and soft tissue structures are arranged and regarded. The models and theories are briefly summarized for a better overview in the following table (Table 4).

The model/the theory as well as a short description concerning function and/or the development basis in each case (so far, the appropriate paper included the information) are shown in Table 4. The different acquisition methods and investigative modalities, as well as limitations associated with cadaver studies and technical aspects of imaging studies, may explain some of the discrepancies among wrist kinematic theories and descriptions [49].

“Although some researchers have postulated kinematic theories that describe the kinetics and kinematics of the normal wrist, there is no universally accepted theory”. [15].

### 4.2. Loads of the Wrist Joint

Load transfer of the wrist is an important factor in wrist joint biomechanics. Different researchers worked on this topic, e.g. stress analysis [58,59,60], force transmission [59,61,62,63,64,65,66], or contact biomechanics [67,68]. Knowledge of force transmission is of importance in understanding the normal joint biomechanics and explaining the pathogenesis of, e.g., osteoarthritis [64] or Kienbock’s disease [61,62].

Force transmission across the wrist in a neutral position and neutral forearm rotation show that approximately 80% of the load is transmitted across the radiocarpal joint [2]. In this case, it is estimated that approximately 45% of the force crosses the radioscaphoid joint and 35% crosses the radiolunate joint [2]. The remaining 20% bears the ulnocarpal joint [2]. The load across the midcarpal joint is distributed at 31% through the scaphotrapeziumtrapezoid joint, 19% through the scapho-capitate joint, 29% through the lunocapitate joint, and 21% through the triquetrohamate joint [2].

Using pressure–sensitive film to define the contact area between bones in the wrist and radius, three distinct regions of contact have been identified in the radiocarpal joint: radioscaphoid, radio-lunate, and ulnolunate [2]. The contact area of the scaphoid and lunate in an intact wrist is approximately 129.8 mm^2^ [67]. Tang et al. found that the scaphoid contact area is in average 77.7 mm^2^ in extension and neutral position and decreased by 42% to 45.2mm^2^ in flexion [67]. Evaluating the contact area within the lunate fossa, the contact area is 63.3 mm^2^ [67]. The lunate contact area averaged 55.7 mm^2^ in flexion and neutral position and increased to 78.6 mm^2^ in extension [67]. The centers of the contact areas change location with changes in wrist position as do the areas of contact [2].

The peak loads across the wrist are quite low compared with those of other joints, ranging from 1.4 MPa (1 MPa = 1 N/mm^2^) to 31.4 MPa [2]. Scaphoid pressure averaged 1.4 MPa and lunate pressure averaged 1.3 MPa and they did not significantly change between wrist positions [67]. There was no significant difference in scaphoid and lunate pressure in all positions [67]. When evaluating force transmission, in extension the scaphoid transmitted 51% of the force and the lunate transmitted 49%, in neutral position 53% and 47%, and flexion 55% and 45%, respectively [2,13,67]. Consistently in load studies, it was found that the location within increased pressure correlated well with areas with degenerative changes [4].

## 5. Conclusions and Future Research

The wrist is a complicated anatomic structure and is considered the most complex joint of the human body. In addition, the motions, load distributions, and biomechanical demands are extremely complex. It evidences equally complicated mechanics to provide a substantial ROM and additionally the load transfer from the hand to the forearm [2,13]. Traditional descriptions of wrist anatomy appear to be inadequate, often conflicting, and sometimes introduce data that seem to fit into no satisfactory logical framework [69]. Physiological wrist function can be impaired very easily by injury or disease. That is the reason why it is important to understand the anatomy and biomechanics of the wrist for an adequate treatment of wrist pathologies.

The wrist ligaments have the responsibility of balancing the constraints to ensure the maintain stability while at the same time, allowing the generous ROM [27]. There exist further functions of the ligaments, e.g., proprioceptive interactions [27]. Moreover, there exists clinical experience concerning ligament reconstruction and the performance of, e.g., partial carpal arthrodesis of wrist bones that still show unpredictable results. This implies the need for a closer investigation into the biomechanics of the wrist joint.

For that, biomechanical modeling could be a possible approach. A unifying model of the wrist biomechanics and functional carpal kinematics, respectively, remains elusively [6]. Existing models come along with a lack. For example, Ruby et al. believe that a PCR/ DCR row model fits the kinematic data better than the column theory of wrist motion [41]. An essential property of a biomechanical wrist model is the possibility of evaluating the influence of, e.g., geometric parameters. Furthermore, with a model, it would be easy to investigate and evaluate the cooperating joint surfaces, of looseness, the surface of the cross-sections, and the lengths of muscles and ligaments on the number of their forces in the function of the hand position toward the forearm and the load conveyed by the hand.

Future research could focus on the integration of material properties, kinematics, and kinetics. Furthermore, additional clinical investigations should continue to attempt for improvement of existing and to develop alternative intervention techniques. Meanwhile, the determination of the efficacy of interventions by carrying out carefully constructed clinical trials and reviews with standardized outcome measures should be a goal. Treating wrist disorders must be based on a proper understanding of the physiological anatomy and biomechanics. The goal must be a patient specific and individualized approach to treat carpal injuries.

## Figures and Tables

**Table 1:** Extrinsic ligaments.

Position	Ligament	Description and Characteristics
Volar radiocarpal ligaments	radial collateral ligament	
	radioscaphocapitate ligament	- creating a sling to support the waist of the scaphoid - preserve when doing PCR−ectomy - acts as the primary stabilizer of the wrist after PRC and prevents ulnar drift
	long radiolunate ligament	- counteracts ulnardistal translocation of the lunate
	radioscapho-lunate ligament	- only functions as a neurovascular conduit - does not add mechanical strength
	short radiolunate ligament	- stabilizes the lunate
Volar ulnocarpal ligaments	ulnotriquetral ligament	
	ulnolunate ligament	
	ulnocapitate ligament	
Dorsal ligaments	radiotriquetral ligament	- referred also as dorsal radiocarpal ligament (DRC) - must also be disrupted for VISI deformity to form (in combination with rupture of the luno-triquetral interosseous ligament)
	dorsal intercarpal (DIC) ligament	
	radiolunate ligament	
	radioscaphoid ligament	

**Table 2:** Intrinsic ligaments.

Position	Ligament		Description and Characteristics
Proximal row	Scapholunate interosseous ligament	dorsal portion	- prevents translation - primary stabilizer of scapholunate joint
		volar portion	- prevents rotation
		proximal portion	
	Lunotriquetral interosseous ligament	dorsal portion	
		volar portion	- strongest part
		proximal portion	
Distal row	trapeziotrapezoid ligament		
	trapeziocapitate ligament		
	capitohamate ligament		
Palmar midcarpal	scaphotrapeziotrapezoid ligament		
	scaphocapitate ligament		
	triquetralcapitate ligament		
	triquetralhamate ligament		

An extensive overview of ligament anatomy and motion behavior is available, e.g., in [11,22,23,27,28,29].

**Table 3:** Muscles of the forearm especially for wrist motion.

No.	Muscle		Origin	Insertion	Function on the Wrist
1	Flexor carpi radialis	(FCR)	Epicondylus medialis humeri	Os metacarpale II	Flexion, Radial Deviation
2	Palmaris longus	(PL)	Epicondylus medialis humeri	Ligamentum carpi transversum (Retinaculum flexorum), palmar aponeurosis	Flexion
3	Flexor carpi ulnaris	(FCU)	Epicondylus medialis humeri, Olecranon	Os hamatum (sesamoid: Os pisiforme)	Flexion, Ulnar Deviation
4	Extensor carpi ulnaris	(ECU)	Epicondylus lateralis humeri	Os metacarpale V	Extension, Ulnar Deviation
5	Extensor carpi radialis brevis	(ECRB)	Epicondylus lateralis humeri	Os metacarpale III	Extension, Radial Deviation
6	Extensor carpi radialis longus	(ECRL)	Crista supracondylaris lateralis	Os metacarpale II	Extension, Radial Deviation

**Table 4:** Summary of the different models and theories.

Model/Theory	Description	Author	Year	Development Basis
Row-theory	2 horizontal rows	Bryce/Destot [55,56]	1896	Anatomical investigations
Column-theory	3 vertical columns	Navarro [51]	1921	-
Screw vice or clamp theory	Mechanical behavior of the wrist	MacConaill [54]	1941	Cadaver and X-ray investigations
Link joint theory	Technical description of the wrist as a linkage system	Gilford [53]	1943	Cadaver and X-ray investigations
Row-Column-theory	Central T-structure in combination with 2 columns	Taleisnik [22]	1976	17 cadaver investigations
Ring-model	The ring structure of the bones including the intrinsic ligamentous apparatus	Lichtman et al. [52]	1981	23 cadaver investigations in combination with 10 clinical examinations
Ovoid/C-shape theory	Midcarpal joints are contained within a midcarpal ovoid, on an axial radiograph of the ovoid, the midcarpal joint displays a C-shaped outline	Moritomo et al. [30,57]	2006	MRI of 24 volunteers
